# Facilitators for Routine Medical Checkups Use from the Perspective of Primary Care Providers: A qualitative study

**DOI:** 10.12669/pjms.41.4.10649

**Published:** 2025-04

**Authors:** Ali M. Alzahrani, Baraa S. Quronfulah, Holly C. Felix, Asim A. Khogeer

**Affiliations:** 1 Ali M. Alzahrani, MSHSA, PhD. Department of Health Administration and Hospitals, College of Public Health and Health Informatics, Umm Al-Qura University, Makkah, Saudi Arabia; 2 Baraa S. Quronfulah, PhD, MPH-HP. Department of Health Promotion and Education, College of Public Health and Health Informatics, Umm Al-Qura University, Makkah, Saudi Arabia; 3 Holly C. Felix, PhD, MPA. Retired Professor, Ponca, Arkansas USA; 4 Asim A. Khogeer, PhD, MSc. Department of Research, PMO, Ministry Branch in Makkah Region and Medical Genetics Unit, Maternity & Children Hospital, Makkah Healthcare Cluster, Ministry of Health (MOH), Makkah 21955, Saudi Arabia

**Keywords:** Facilitators, Routine Checkups, Routine Medical Checkups, Saudi Arabia

## Abstract

**Objective:**

To identify facilitators to routine medical checkups (RMCs) use among Saudi adults from the perspectives of primary care providers (PCPs).

**Methods:**

A qualitative phenomenological study design was conducted at five primary health care centers (PHCs) in Makkah, Saudi Arabia. Data was collected between December 2020 and February 2021 using semi-structured interviews with 19 PCPs. Descriptive statistics were performed to characterize participants, and a direct content analysis was conducted to identify major themes.

**Results:**

A number of factors were identified as facilitators for RMCs use among Saudi adults. Those facilitators were classified into three main themes related to patients, providers, and healthcare systems. Patients’ knowledge about the importance of RMCs and their awareness of the service availability were identified as key facilitators under patients-related theme. Providers’ knowledge, skills, and awareness of RMCs were identified as facilitators under provider-related themes. Lastly, improving the supply of providers and enhancing access were the facilitators identified under the healthcare system-related theme.

**Conclusion:**

This study provided insights into the facilitators for RMCs use among Saudi adults from the perspective of PCPs. This could contribute to the knowledge and inform future research, policy, and decision-making related to increasing the use of RMCs among Saudis.

## INTRODUCTION

A routine medical checkup (RMC) is a visit to a primary care doctor to maintain health and wellness without having a specific illness or complaint. At the time of the RMC, medical examination and laboratory investigation can be reserved for non-complaining individuals.^1^ RMC is helpful and effective in discovering new disease cases and preventing major complications through early intervention.^2^

Globally, the frequency of RMC varies from country to country. In Germany, the percentage of RMCs is 50.8% for men and 49.8% for women.^3^ While in Japan, only 38.4% of middle-aged adults and elders have RMCs.^4^ In Vietnam, previous studies specified that even in urban areas, the proportion of people having RMCs was only 51.2%.^5^ In Saudi Arabia, the proportion of people aged 30 and over with RMC is 34.3%,^6^ even though individuals who are registered at healthcare clinics can get medication and consultation without charge.^7^ Yet, variation in the understanding of the importance of RMCs in Saudi Arabia is controversial and not fully described.^6^,^8^,^9^

In addition, several factors have shaped individuals’ attitudes toward going for RMCs, and different barriers have been reported in many studies.^6^,^10^,^11^ Examples of barriers to the use of RMCs include lack of time, perceived benefits of RMCs, and health concerns.^6^,^10^,^11^ Facilitators to RMCs (factors that encourage people to have RMCs) vary from place to place. Having health insurance, advanced age, demographic and socio-economic class status, mass media, and excellent community support are factors that facilitate the use of RMCs.^12^-^15^ Other factors that may facilitate RMCs are an individual’s belief in treatment and a patient’s trust in their primary care providers (PCPs).^16^,^17^ Previous studies suggested that local and international communities have an appropriate level of knowledge concerning the importance of RMC.^18^ However, some studies attributed the general care practices as a negative contributor toward individuals’ RMCs.^6^,^7^

Lastly, free-of-charge primary health care services in Saudi Arabia make RMCs accessible to people.^19^ However, previous research showed that Saudis are not using this free service.^6^ A recent national survey showed that almost three-quarters of Saudis have never had an RMC.^16^

Previous research investigated the barriers and facilitators to RMCs from the perspectives of healthcare service users without considering the PCPs’ perspectives. Thus, this study aimed to provide an in-depth understanding of the facilitators to RMC use among Saudis from the PCP’s point of view.

## METHODS

A qualitative phenomenological study design was adopted at five primary health care centers (PHCs) in Makkah, Saudi Arabia between December 2020 and February 2021. Data collected using semi-structured interviews was divided into two parts. The first part involved introducing the research topic to interviewees by stating the meaning of RMCs discussed during the interview. The guiding interview statement was: “We are interested in hearing your opinions regarding the low use of routine health checkups by Saudi adults. By routine health checkups, we mean a general physical exam, not an exam for a specific injury, illness, or condition.” Then, the interviewers asked participants about their perspectives on barriers and facilitators for RMCs using several open-ended probing questions. Interviewers also asked participants to clarify comments during the interview using spontaneous questions. The second part of the interview involved collecting participants’ sociodemographic data including: age, sex, nationality, duration of practice as PCPs in years, and medical specialty. Similarities in the methodology may be found in another publication generated from this project.

The target sample size was 20 participants, which is within the recommended sample needed for qualitative phenomenological studies.^20^ Participation eligibility criteria were:

1. Being a PCP who works as a general practitioner, family or community medicine specialist, or consultants;
2. Working at one of the five selected primary health care centers (PHCs) in Makkah, Saudi Arabia that was operated by the Ministry of Health (MOH). The authors decided the selection of the PHCs to cover Makkah geographic area and the fact that RMCs were available at those PHCs.

### Ethical Approval:

This study was conducted following the Helsinki Declaration. The study protocol was reviewed and approved by the Makkah Health Affairs General Directorate’s Institutional Review Board (Approval Number: H-02-K-076-1120-405, dated Novmber 23, 2020).

The researchers (BSQ and AMA) recruited participants. A recruitment letter was prepared by MOH staff and sent to the selected PHCs managers. The letters described the aim of the study and facilitated researchers’ visits to interview PCPs. Researchers (BSQ and AMA) called the PHCs 2-3 days after the recruitment letters had been received and invited PCPs to participate in the study. After explaining the purpose of the research to prospective PCPs, those who agreed to participate in the study were asked for a convenient time to conduct the interview.

Two researchers (BSQ and AMA) conducted interviews between December 2020 and February 2021. Of the 19 PCPs interviewed, 17 were interviewed in a quiet room in their PHCs. Two PCPs preferred an online interview using Cisco WebEx meeting software.

### Procedure:

At the beginning of the interview, the researchers provided the study information sheet to PCPs and gave them time to read and understand the study purpose, procedure of participation, data privacy, and the confidential nature of the study. Participants were encouraged to ask any questions before signing the MOH informed consent form. The researchers started the recorder and began the interview using the abovementioned interview guide. At the end of the interview, the researchers thanked the participants for their time and efforts. Participants’ contact information was requested from participants if they were willing to be contacted if needed by the researchers for response validation.^21^ The interviews took about 20-30 minutes. Researchers and participants wore face masks and maintained at least one-meter distance between them during in-person interviews in order to protect against COVID-19. No remuneration was provided for participation.

### Data Analysis:

Descriptive statistics (e.g., mean, standard deviation, percent) were calculated using Stata/SE 16.0 (StataCorp, College Station, Texas, USA) to profile the study participants. Interview audio recordings were transcribed verbatim for direct content analysis to identify major themes. Direct content analysis is useful for expanding understanding of specific situations, such as the use of RMCs.^22^,^23^ Transcripts were read by researchers (AMA and BSQ) for overall content and then coded in ATLAS.ti 22.0 (ATLAS.ti Scientific Software Development GmbH, Berlin, Germany) using predetermined and new codes developed as the coding process was undertaken. After independent coding, two researchers (AMA and BSQ) together reviewed the coding, discussed differing coding, and agreed on final codes. Finally, all researchers (AMA, BSQ and HCF) identified major and minor themes from the final coded material, discussed the themes and their implications, and made recommendations. Qualitative data are reported in aggregate form, with quotes masked to protect the participants’ identity.

## RESULTS

The sociodemographic characteristics of the 19 participants is shown in Table-I. The mean age of the participants was 36.32 years (standard deviation (SD) 1.75 years), and the average length of practice as a PCP was 7.35 years (SD=1.32). More than half (52.63%) of the participants were male, and the majority (84.21%) were from Saudi Arabia.

**Table-I:** Socio-demographic Characteristics of the Study Sample (n=19).

	Mean	SD
Age, in years	36.32	1.75
Years in practice as a primary care provider	7.35	1.32
	N	(%)
*Gender*		
Female	9	47.37
Male	10	52.63
*Nationality*		
Saudi	16	84.21
Non-Saudi	3	15.79

SD: Standard Deviation.

Factors identified by providers as facilitators for RMCs use were classified into three main themes: Patient-related facilitators, Provider-related facilitators, and Healthcare system-related facilitators. Under each theme, important subthemes were identified and defined.

### Theme 1: Patient-related facilitators

Providers identified several facilitators related to patients’ getting RMCs. These facilitators fall under two subthemes: patients’ health literacy (knowledge) and patients’ awareness of RMC service.

### 1.a. Patients’ health literacy (knowledge)

Focusing on increasing patients’ knowledge about the importance of RMCs was considered as a facilitator by participating providers. One participant said: “We have to educate [the] general population about the need for this such screening at our level at the primary care.” (PCP6). Another participant said: “Make orientation to patient and to general population about the importance of coming for routine checkups.” (PCP15). Additionally, two other participants indirectly discussed the importance of increasing patients’ knowledge about the importance of RMCs when they provided examples of screening for some medical conditions: “… tell patients and advise them about hypertension, diabetes, and obesity.” (PCP18), and “… tell people about the influenza vaccine and its importance especially this year, and consequently there was a high response from people to come and get the influenza vaccine.” (PCP2).

Providers identified health promotion and health education services, including health coaching, as a key facilitator to improve patients’ knowledge about the importance of RMCs. One participant said: “Health promotion and health education is the biggest facilitator to improve the knowledge and awareness about this investigation to improve the health of the population and community.” (PCP16), Another participant said, “health educators and health coaches within the primary care centres could play a major role” as “health education is the most important key in any management plan.” (PCP12). Other participants discussed the need of having health educators at PHCs and the fact that health promotion and education graduates could be a good fit for this role as opposed to physicians. One participant suggested: “more activities for health education and promotion clinic” would be good (PCP2). Another participant noted, “I think [there’s] no need to put one doctor in this clinic, any health provider like those we have here in our primary health care centre … some students or interns from health education college, they are here talking with patients doing for them labs and something like this, I think those type of people can help with this” and “I think many students graduated from those colleges are sitting at home and we can take those people and put them in the primary health care centres to help us to do the best.” (PCP13). Another participant discussed the need of having male and female health educators in each centre to account for Saudi social norms: “So, if we like work on having the educators in each centre, and even more than one, male and female if someone’s not comfortable talking to the other gender. So, if we influence this from the Ministry of Health, and they make primary health care centres have one or two of them, I think it’s going to be a game changer.” (PCP12).

### 1.b. Patients’ awareness of RMCs

Providers highlighted the need to increase patients’ awareness about the availability of RMCs at primary health care centres. One participant said: “… we need to identify this service more” (PCP7). Another participant discussed that there is lack of awareness of the services among patients: “… and most of people what I believe they did not know about it [routine checkups]. So, we have to raise a voice for this, for these services [routine checkups].” He added: “… I think we have to do some announcement for these services [routine checkups].” and “So, we need to do advertise our services, including, or especially this periodic health examination.” (PCP11). On the other hand, one participant discussed that some patients became aware of the role of the family physician in preventing illness. People become aware that primary health care centres are for prevention and about the role of family physicians.” (PCP8).

As for the best ways to increase patients’ awareness of the availability of RMCs at primary health care centres, participants proposed using mass media campaigns especially social media to reach larger populations, saying. “Maybe [we] need media, put this idea in the media, the social media, something like this to make open mind of people in the culture about this [routine checkups].” (PCP5), and “Nowadays, all of us are using social media for education. Like influenza vaccine is increasing because there is an improvement in the awareness. So, all people, adolescents, adults, and young people are using social media which is an important factor.” (PCP8), and “Using the media, the internet, and social applications…to educate people and reach a larger population.” (PCP16), and “We have to prepare some short videos. We have to do it in social media. We have to invite some influencer to promote for these services, because it is very important.” (PCP11). Another participant discussed other opportunities to raise awareness of RMCs among patients, like distributing advertisement and flyers at gatherings in the community. Some identified examples were schools and Mosques, where people drop off their children every day at school and go five times a day to Mosque for praying: “we can put this advertise[ment] for example in the Mosque entrance, at the schools’ gates like that this advice for routine checkups to be promoted and to have a good health.” (PCP9).

### Theme 2: Provider-related facilitators

Participants also identified provider-related factors that facilitate the uptake of RMCs. These facilitators were mainly related to providers’ knowledge and skills about RMCs.

### 2.a. Providers’ Knowledge/Skills

Participating providers discussed the need to train providers at PHCs and improve their knowledge about RMCs: “We need more staff training.” (PCP10), and “We need to educate … our staff either doctors or nurses or even lab workers to promote this practice of our routine checkup.” And “To practice more because the problem mainly from for example our poor practicing people are afraid to perform this [routine checkups].” (PCP9). Another participant highlighted the need to conduct research and disseminate the findings among providers to increase their awareness about the low use of RMCs which may encourage them to raise this issue to decision makers at the MOH: “We can do research like you are doing, so we know how the percentage of people who are not aware about these screening. … for example, low percentage of people seeking screening. This will raise the awareness more regarding us [providers] or for the high authority, so if we write recommendations for the high authority, so have evidence like what you are doing now.” (PCP6).

### Theme 3: Healthcare system-related facilitators

Providers also reported factors related to the healthcare system that might facilitate the use of RMCs. These facilitators can be classified into two main subthemes: supply and access.

### 3.a. Supply

Providers highlighted two main facilitators under this subtheme. The most frequently mentioned facilitator was activating a specific clinic for RMCs at PHCs. For example, PCP10, said “We need a separate clinic with a sign, so it’s really for checkup …. just for screening, [the clinic] has its own doctor and nurse.” Another provider said, “… if we can put one clinic with someone staying in this clinic only for a routine checkup, this will be great.” (PCP13). Several providers believed that setting up a specific clinic for RMCs would facilitate the uptake of RMCs by reducing the waiting time, saying “We might specify one clinic for annual exam, so no waiting time.” (PCP17) and “Maybe, we’ll put one clinic for routine checkup to deal with that busy clinic [other/regular clinics within the PHCs] …” (PCP4).

The second facilitator identified under this subtheme was understaffing at PHCs. As an example, PCP10, said “We need more staff.” … “… we need more health educator and a primary care health educator, so they will help us in this program [routine checkups]”. Another provider, said “Increase the staff in primary health care centers as I said increase the capacity.” (PCP15).

### 3.b. Access

Under the access subtheme, providers identified more facilitators including reaching out to people, attracting and incentivizing them, establishing a national RMC program, and building an appointment system for RMCs. Different ways have been suggested to reach out to people to facilitate the uptake of RMCs. For example, a provider said “We got about the opportunity to reach, we will reach the patient or the client, we will not wait [for] the client to come to us. We have to go to them, so we can send them SMS messages, or we can contact them” (PCP11), while another one said, “Maybe we tell them, we text them” (PCP18). Two other providers suggested using the technology to reach out to people, saying “So, we need to have some application for that [routine checkups], because you know these examinations or periodic health examination, that means I have to see these clients many times.” (PCP11) and “We can make it online.” (PCP13). One provider suggested reaching people physically, saying “We can go to the patients in their homes and do a checkup and orientation like this … or we bring them to the center to make examination by helping in transportation” (PCP15). Also, one provider mentioned that MOH should attract people and incentivize them for doing RMCs, saying “MOH must do screening programs, advertise and give advantages … you attract people first and you can give them what you want education or screening.” (PCP3).

To increase access and facilitate the uptake of RMCs, several providers also emphasized the need for establishing a national RMC program and scheduling system. For example, PCP14 said “We can start what was there before, a checkup (periodic health examination program) …. starting periodic health examination or prevention program, this will be cost effective, will decrease the morbidity of disease, bad prognosis, ….” Another provider said, “Maybe if Ministry of Health adopt[ed] a program specializing in this service [routine checkup] ….” (PCP7). A provider suggested a timeframe for RMCs, saying “I think this [routine checkups] may need to make schedule like vaccination schedule …., when 25 years old coming to checkup, after 10 years like this, and then after five years, every six months, every two months like this” (PCP5).

Finally, several providers also emphasized the need for establishing a scheduling system, saying: “Everything will go well, but we need to make an appointment, the appointment is everything. If there is an appointment for screening, it is going to be great also” (PCP19) and “…. and they [patients] need to have scheduling for screening and another appointment at another separate clinic ….” (PCP10).

## DISCUSSION

In this study, we tried to identify facilitators to RMC use among Saudis from the perspective of PCPs. The main themes included factors related to patients, providers, and healthcare systems. Considering the high prevalence of noncommunicable diseases and the low uptake of RMCs among the Saudi population, understanding factors that facilitate the use of RMCs may alleviate current barriers and increase the use of RMCs which help in detecting diseases in early stages when treatments are effective and can ultimately improve populations’ health. The three identified facilitator themes (patient-related facilitators, provider-related facilitators, and healthcare system-related facilitators) are discussed below.

PCPs identified patients’ health literacy (knowledge) related to RMCs and their awareness of the availability of RMCs as important facilitators. PCPs suggested if patients understood the importance of RMCs and were aware of the availability of RMCs at PHCs, they would be more likely to get RMCs. There are multiple ways in which to educate the general population on health-related topics. PCPs suggested that health educators and health coaches working within the PHCs could play an important role with educating patients about RMCs.

Health coaches and health educators have been used to help patients improve their health knowledge and change health behaviors. For example, a recent Saudi Arabian study found that a 12-week diabetes self-management intervention using health coaches significantly lowered HbA1c levels among diabetic patients compared to their peers who received standard care.^24^ Using health coaches to provide education about RMCs and their importance may also be effective in facilitating uptake of RMCs.

PCPs mentioned using print media in the form of flyers which would be posted or circulated at community locations where large numbers of people gather, such as at schools and Mosques. Such public health education campaigns have been shown to be effective in increasing knowledge about cancer screenings among female Saudi university students.^25^ PCPs also mentioned using social media applications to circulate information and short videos which could spread relevant information about the importance of RMCs and where they can be had. Systematic reviews of social media applications being used in health promotion indicate they can be effective in positively changing heath behaviors.^26^ Participants of one study of a Twitter-based breast cancer support program found that the social media application was effective in increasing knowledge about breast cancer and decreasing cancer related anxiety.^27^ Moreover, text message reminders of breast cancer screening appointments significantly increased appointment attendance.^28^ The Saudi public may be very receptive to this type of intervention given that more than half of Saudi adults who participated in a survey study regarding RMCs suggested social and traditional media outlets could be a good mechanism for educating the public about RMCs.^6^,^15^

PCPs identified providers’ knowledge, skills and awareness of RMCs as facilitators to the use of RMCs. Previous research has reported similar findings. A study from India evaluated factors affecting preventive health checkups and found that reliable providers, doctors who can assess preventive health, as a significant factor that enables the increased use of preventive health checkups.^29^ Another study by Bowser et al. (2017) reviewed the literature to identify facilitators to the use of breast cancer screening and found that informative providers, who were knowledgeable about the screening services, were significant facilitators to implement and increase the use of screening services.^30^ Therefore, increasing providers’ knowledge about RMCs and its availability in the system could be an effective intervention to increase the use of RMCs.

Providers suggested making changes to the healthcare system in Saudi Arabia as a way to facilitate use of RMCs. These changes related to improving the supply of providers to perform RMCs and developing systems to facilitate access. Specifically, providers suggested developing specialized or dedicated clinics to perform RMCs, noting that such clinics could reduce the wait time for appointments. An intervention in the United Arab Emirates (UAE) created specialty appointment-based clinics to treat patients with diabetes and hypertension. Providers received training specific to diabetes, hypertension, dyslipidemias and cardiovascular risk, including use of evidence-based treatment guidelines for these conditions. Patients using these specialty clinics saw significant improvements in blood pressure and HbA1c.^31^ Although these specialty clinics did not perform RMCs, they do provide evidence that dedicated staff focused on specific conditions can improve patient outcomes. Thus, clinics dedicated to RMCs may increase awareness of RMCs and increase the efficiency in which they are performed. As was done in the UAE study, a dedicated RMC clinic should be pilot-tested to assess its performance before wider roll-out of such clinics occurs.

In terms of access, providers suggested using digital technologies to facilitate use of RMCs. One suggestion was for the development of an RMC application. Such an application could provide information about RMCs to increase understanding of them as well as to facilitate access to RMC appointments. In 2021, the Saudi Arabian MOH launched the “Know Your Numbers” campaign to encourage the public to monitor four vital signs (blood sugar, blood pressure, waist circumference and body mass index) with those vital signs being available through *‘the Sehhaty’* application. An early evaluation, conducted within six months of the launch of the campaign, found that only about one-third of the survey study participants had heard of the “Know Your Numbers” campaign and less than 5% actually participated in the campaign.^18^ Therefore launch of an RMC oriented application should be supplemented with a strong outreach program to facilitate its use and an evaluation plan to determine its effectiveness.

Providers also suggested home visitation programs to perform RMCs. Home visitation programs have been used for medical personnel to check on a variety of patients. For example, a systematic review and meta-analysis found that home visitation programs of patients with hypertension were successful in lowering systolic and diastolic blood pressure and waist circumference although there were no improvements in body mass index, weight or blood lipids.^32^ However, home visitation programs can be more expensive that standard care,^33^ which raises concerns about the financial viability of such a program to facilitate rates of RMCs.

The use of a national RMC program and scheduling system was also suggested. A systematic review of such appointment systems indicated they can decrease missed appointment rates, staff labor, and waiting times as well as improve patient satisfaction.^34^ The Saudi Arabian MOH launched *‘the Mawid’* application which allows patients to schedule appointments at primary care hospitals. An evaluation of the application indicated high levels of overall patient satisfaction.^35^ Although ‘*the Mawid’* application is used for all primary care hospital appointments; a similar application could be developed which scheduled appointments specifically for RMCs.

### Limitations:

First, the recruited sample came from five PHCs located in Makkah city, which may have affected the external validity of the study, limiting the generalizability of the findings. However, the results have highlighted important facilitators, from the perspective of PCPs, to increase the uptake of RMCs. Second, the interviews were conducted during working hours (when PCPs are busy with their clinics and patients), which may affect their willingness and limit their opportunity to participate in this study. However, remote/online interviews were made available to alleviate the challenge of the PCPs’ time availability and reduce the risk of selection bias. Despite these limitations, a particular strength of this study is its contribution to the Saudi health literature. To the best of our knowledge, past literature is lacking research on facilitators to the RMCs use among Saudis from the perspective of PCPs.

## CONCLUSION

This study has identified and highlighted some patient-related, provider-related and system-related facilitators that enable Saudis to use RMCs. This contributed to the dearth of the literature; provides insights on facilitators to RMC use and laid the foundation for future research that can inform future policy and decision-making related to increasing the RMC use among Saudis. Future research with a representative sample is recommended to examine a wider variety of experiences and perspectives regarding the facilitators to the RMC use among Saudis.

### Authors’ Contributions:

AMA, BSQ and HCF: Conception and design.

AMA, BSQ and AAK: Acquisition of data.

AMA, BSQ: Analysis and interpretation of data.

All authors have contributed to drafting and revising the article, approved the final version to be published, and agreed to be responsible and accountable for the accuracy or integrity of the work.
